# Hemispheric Asymmetry in New Neurons in Adulthood Is Associated with Vocal Learning and Auditory Memory

**DOI:** 10.1371/journal.pone.0108929

**Published:** 2014-09-24

**Authors:** Shuk C. Tsoi, Utsav V. Aiya, Kobi D. Wasner, Mimi L. Phan, Carolyn L. Pytte, David S. Vicario

**Affiliations:** 1 Biology Department, The Graduate Center, City University of New York, New York, New York, United States of America; 2 Psychology Department, Rutgers, The State University of New Jersey, Piscataway, New Jersey, United States of America; 3 Psychology Department, Queens College, City University of New York, New York, New York, United States of America; Claremont Colleges, United States of America

## Abstract

Many brain regions exhibit lateral differences in structure and function, and also incorporate new neurons in adulthood, thought to function in learning and in the formation of new memories. However, the contribution of new neurons to hemispheric differences in processing is unknown. The present study combines cellular, behavioral, and physiological methods to address whether 1) new neuron incorporation differs between the brain hemispheres, and 2) the degree to which hemispheric lateralization of new neurons correlates with behavioral and physiological measures of learning and memory. The songbird provides a model system for assessing the contribution of new neurons to hemispheric specialization because songbird brain areas for vocal processing are functionally lateralized and receive a continuous influx of new neurons in adulthood. In adult male zebra finches, we quantified new neurons in the caudomedial nidopallium (NCM), a forebrain area involved in discrimination and memory for the complex vocalizations of individual conspecifics. We assessed song learning and recorded neural responses to song in NCM. We found significantly more new neurons labeled in left than in right NCM; moreover, the degree of asymmetry in new neuron numbers was correlated with the quality of song learning and strength of neuronal memory for recently heard songs. In birds with experimentally impaired song quality, the hemispheric difference in new neurons was diminished. These results suggest that new neurons may contribute to an allocation of function between the hemispheres that underlies the learning and processing of complex signals.

## Introduction

Converging evidence suggests that new neurons formed in adulthood contribute to the formation of new memories [1], [2]. Learning and memory formation have also been shown to be functionally lateralized such that corresponding regions in the two hemispheres are not purely redundant, but instead provide specialized processing of related tasks and may also show anatomical differences [3]. One prominent example of cerebral lateralization is hemispheric dominance for the production and perception of language, but many other functions and brain regions also show lateral differences in humans and animals [4], [5], [6], [7], [8], [9], [10], including regions that receive new neurons throughout adulthood [8], [9], [10], [11], [12], [13]. Despite this, the role of new neurons in differential contributions to behavior by the two hemispheres is unknown [14], [15]. The present study combines cellular, behavioral, and physiological methods to address this gap in knowledge.

We quantified new neurons (labeled during cell division 1 month earlier) in both hemispheres of the caudomedial nidopallium (NCM), a songbird auditory forebrain area, and compared new neuron lateralization with individual differences in the quality of vocal learning and with the strength of neuronal memory for song. The contribution of hemispheric asymmetry to sensorimotor learning can be assessed in this model because songbirds learn their communication signals by imitating a tutor, as humans do [16], and songbird brain areas that subserve motor and sensory aspects of song learning and production continuously receive new neurons throughout adulthood [17]. Moreover, hemispheric differences in electrophysiological responses in NCM, which responds preferentially to conspecific songs, have been shown to require experience with conspecific vocalizations during development [12]. It has been proposed that the anatomical structures associated with vocal learning show both an evolutionary convergence and share cell type homologies between avian and human brains [18], [19], [20], [21]. In a striking parallel with lateralized speech production and perception, songbird vocal processing areas are functionally lateralized, as demonstrated by behavioral, electrophysiological, fMRI, and immediate early gene studies [7], [12], [13], [22], [23], [24], [25], [26], [27], [28], [29], [30].

Our results demonstrate an intriguing hemispheric asymmetry in the numbers of new neurons in the auditory forebrain that is correlated with both behavioral and physiological measures of learning and memory. Furthermore, this correlation is disrupted by lesions that degrade normal vocalizations. This suggests that these new neurons may contribute to, or reflect, a division of function between the hemispheres that provides the capacity for learning complex vocal signals that are important for communication.

## Materials and Methods

### Housing

All procedures were approved by the Institutional Animal Care and Use Committees at Rutgers University and Queens College, CUNY. Male zebra finches (*Taeniopygia guttata*) used for this study (n = 31) were bred and raised in family cages with one male-female pair and a single clutch. Family cages were communally housed in large rooms either at Rutgers University or Queens College, CUNY. Most subjects remained in family cages until>90 post-hatch day (phd) at which time they were housed in all male groups of 8–10 (n = 27). The others (n = 4) were transferred to sound attenuated boxes at 15 phd with their mother and siblings and then were housed individually beginning at 35 phd. These individually housed birds could elicit playback of a tutor song by pecking either of two keys located on the rear wall of the cage for a maximum of 20 playbacks/day (as in [31], [32]). We found fewer new neurons in NCM of singly housed birds compared with group housed birds (consistent with [33]); however, differences were not significant and therefore data from the live-tutored and computer-tutored groups were combined.

### Sample sizes and bird ages

New neuron numbers in left and right NCM were calculated for 31 birds. Subsets of this group were used for additional experiments and analyses as follows. We recorded songs and tutor songs from 21 birds, which were used to compare song copying and song stereotypy with numbers of new neurons. Neurophysiological recordings in NCM were conducted in 20 birds in order to compare the strength of 20-hour song memories (defined below in *Neurophysiological recording in NCM*) with numbers of new neurons. Because genetic or environmental factors were shared by siblings and thus their data were not fully independent, we averaged song copying and electrophysiology values for each sibling group, which resulted in a final sample size of 14 birds with both song copying and electrophysiological measures. This group included 5 sets of (2 or 3) siblings tutored by a live male, 6 unrelated birds tutored by different live tutors, and 3 individual males who received song playbacks in response to self-initiated key pecks. Song recordings were not obtained for one of our computer-tutored birds, which was nonetheless used for new neuron data.

In a different set of birds, the vocal nerve innervating the syrinx was sectioned unilaterally to disrupt song production (n = 6 right side nerve cut, n = 7 left side nerve cut, n = 7 control), described further below. The control birds were included in the set of 31 birds in which we compared new neuron numbers in left and right NCM. Song learning and electrophysiology were not assessed in either of these groups of nerve cut birds or in their controls.

All birds used for new neuron counts received bromodeoxyuridine (BrdU) to label dividing cells between the ages of 82–480 phd (approximate mean bird age = 213 days, SEM = 20.6; ages were estimated for 4 birds). New neuron proliferation and/or survival decreases with age in several systems ([34], [35], [36], [37] c.f., [37]). Because of the large age range of birds used for cell birth dating, we explored the possible contribution of age to our results. We did not find correlations between bird age at BrdU injection and any of the values of new neuron numbers we assessed: numbers of new neurons in left or right NCM, total number of new neurons combined across both hemispheres, or new neuron asymmetry index (NAI, defined below in *Neuron asymmetry index*, P>0.05 for all; see [34], [37], [38] for age-related declines in new neurons added to HVC). There were also no significant correlations between bird age and measures of 20-hr memory (P>0.05).

### Timeline

**Figure 1:**
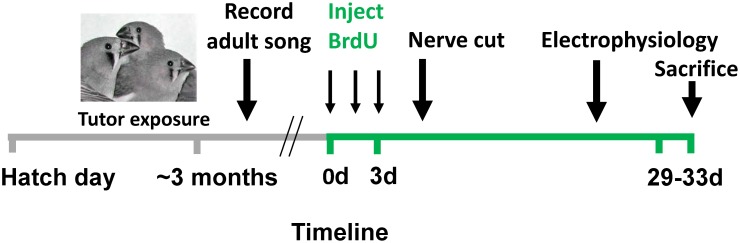
Experimental timeline. Juvenile male zebra finches were exposed to adult male song throughout development (d = day from first BrdU injection). BrdU injections were administered 3x/day for 3 days. Four weeks after the last BrdU injection, multi-unit activity was recorded simultaneously from multiple electrodes placed bilaterally in NCM of awake, restrained birds in response to playback of auditory stimuli. Not all birds were used for all data sets: See text for sample sizes in each experimental condition.

Nerve cuts were made 6–9 days after the first BrdU injection. Electrophysiological recordings were made 26–30 days after the first BrdU injection. Birds were sacrificed by perfusion 31±2 days after the first BrdU injection and thus labeled neurons quantified in NCM (given 3 days of injections) were 29–33 days old (Fig. 1). Because cell death caused by electrodes can potentially alter subsequent neural proliferation, recruitment, and new neuron survival in NCM, we sacrificed birds soon (1–4 days) after conducting electrophysiology, thereby minimizing the impact of tissue damage on new neuron recruitment. Moreover, because electrodes could directly damage BrdU-labeled neurons already in NCM, we avoided counting labeled cells in sections within 100 microns of electrode tracts.

### Bromodeoxyuridine labeling

All birds received intramuscular injections of bromodeoxyuridine (BrdU; 78 µl of a 10 mg/ml solution in 0.1 M Tris buffered saline, pH 7.4, Boehringer Mannheim, Indianapolis, IN) 3 times a day for 3 consecutive days to label mitotically active cells.

### Immunohistochemistry

Birds were perfused with saline followed by 4% paraformaldehyde as in [39]. Brains were serially dehydrated in ethanol and embedded in polyethylene glycol (PEG, M.W. 1500) and stored at 4°C. Tissue was cut sagittally at 6 µm and every 8^th^ section was mounted onto Superfrost Plus slides in 3 series, each offset by one section, and stored at −20°C. Sections were double-labeled with antibodies to BrdU (to identify new cells) and the neuron-specific proteins Hu or NeuN (to identify new cells as neurons). Briefly, tissue was brought to room temperature, then immersed in 90°C citrate buffer for 10 min, rinsed in phosphate buffer (PB), brought to 37°C in PB, and placed in 2.5% pepsin in 0.1 N HCl at 37°C for 3 minutes. Tissue was then rinsed in PB and blocked in 10% normal donkey serum and 0.3% Triton X in PB for 1 hour. This was followed by incubation overnight at 4°C with primary sheep anti-BrdU in block (12.5 µg/ml, Capralogics, Hardwick, MA). Tissue was then rinsed in PB, blocked with an avidin and biotin blocking kit following manufacturer’s instructions (Vector Laboratories), followed by a 2 hour incubation in biotin-conjugated donkey anti-sheep IgG (1:200, Millipore). Tissue was washed in PB and exposed to streptavidin conjugated to Alexa 488 (1.25 µg/ml, Molecular Probes) for 1 hour. All incubations with fluorochromes were conducted in the dark. Tissue was rinsed in PB, blocked for 1 hour, and exposed to either mouse anti-Hu primary antibody (10 µg/ml in block, Hu MAB16A11, Life Technologies) or mouse anti-NeuN antibody (10 µg/ml in PB, MAB377, EMD Millipore) overnight at 4°C. Tissue was rinsed in PB, then incubated in donkey anti-mouse IgG conjugated to Cy-3 (6.25 µg/ml, Jackson ImmunoResearch) for 1 hour to visualize Hu or NeuN. Tissue was washed in PB, dehydrated in ethanols, immersed in xylenes, and coverslipped with Krystalon. All washes in PB were 3 times for 10 minutes each.

### Tracheosyringeal (NXIIts) nerve cut

In an additional set of birds, the vocal nerve innervating the syrinx was sectioned unilaterally to disrupt song production (right side NXIIts-cut n = 6; left side NXIIts-cut n = 7, control n = 7). Birds were anesthetized with ketamine (0.03–0.05 mg/g weight of bird) and xylazine (0.06 mg/g), 6–9 days after the first BrdU injection. Following [25], an incision was made in the skin along the neck and the NXIIts nerve was isolated from the surrounding tissue. For control birds, the nerve was gently manipulated in situ to mimic dissection, but was not cut. For experimental birds, the nerve was cut in two places and the intervening 2–3 mm section was removed to discourage regrowth. The skin was then sutured in both sham and nerve-cut surgeries. Birds were kept isolated postsurgically for several hours and then replaced in their respective home cages. Birds in this experiment were housed together in like-treatment cages (controls, left side NXIIts-cut, or right side NXIIts-cut). As described above, perfusions were conducted 31±2 days after the first BrdU injection.

### Analysis of new neuron densities

**Figure 2:**
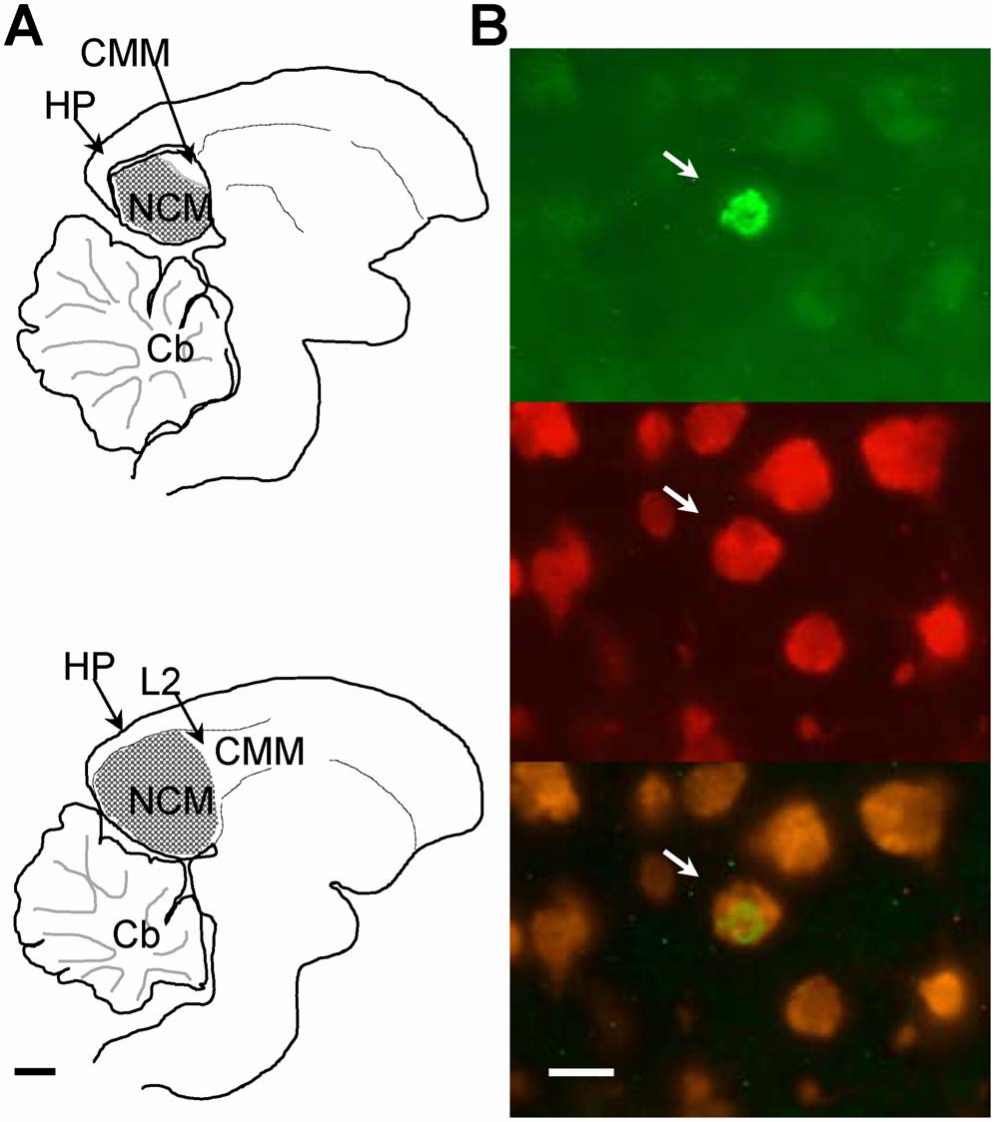
New neurons in NCM. (**A**) Medial (top, ∼170 µm from the midline) and lateral (bottom, ∼500 µm from the midline) sections of NCM (stippled area), showing the region where new neurons were quantified. Abbreviations: Cb, cerebellum; CMM, caudomedial mesopallium; HP, hippocampus; L2, Field L2; NCM, caudomedial nidopallium. Scale bar = 1 mm. (**B**) Photographs of a 1-month-old new neuron in NCM (arrow) in the same field of view showing a BrdU+ nucleus (top), Hu+ neuronal cytoplasm (middle) and co-localization of both markers (bottom). Scale bar = 10 µm.

Histological sections that had been reacted to reveal labeled neurons were analyzed by an observer blind to the hemisphere and the bird’s identity. The boundaries of NCM (as defined in [39]), were traced in sagittal sections with darkfield optics, using Neurolucida and Lucivid (Microbrightfield, Inc., Colchester, VT). New neurons were visualized using fluorescein isothiocyanate (FITC) and rhodamine filters and a dual FITC/rhodamine filter. Labeled neurons in NCM were counted in 10–12 sections collected at regular intervals from ∼170 to 500 µm lateral to the midline of each hemisphere (Fig. 2). Sections were matched along the medial-lateral extent by landmarks (Field L, dorsal arcopallial lamina, and HVC) across hemispheres and across birds. None of the sections sampled were within 100 µm of an electrode track from electrophysiological recordings. The overall density of double-labeled cells per mm^3^ in NCM for each hemisphere was calculated by dividing the sum of double-labeled cell counts by the sum of the volumes sampled. The total volumes of NCM sampled did not differ between left and right hemispheres (paired t-test, t = 0.038, P = 0.707). The difference in new neuron densities between the two hemispheres was evaluated using a paired t-test (two-tailed). The relative difference in new neurons added to the left and right hemispheres was calculated as a percentage by subtracting the new neuron density (counts per mm^3^) in the right hemisphere from the density in the left and dividing by the mean number of new neurons in both hemispheres.

We did not correct for cell splitting in our counts of new neuron densities or calculation of Neuron Asymmetry Index (below) because we were primarily interested in relative differences in new neuron numbers between the hemispheres and between individuals [40], [41]. However, we did correct for cell splitting in estimating the true number of new neurons added to NCM per day. First, we estimated the true value of new neuron counts from our sampled counts using the Abercrombie correction (described in [41]). The mean diameter of BrdU-labeled nuclei we measured and used in the Abercrombie correction was 6.56 microns. We then calculated the estimated number of new neurons added to NCM per mm^3^ per day as follows. BrdU is available for 30–60 minutes after i.m. injection [42]. We used the more conservative end of this range (60 min) and calculated that 3 injections per day over 3 days (9 injections) labeled cells dividing over a total of 9 hours. We divided our corrected value of new neuron counts in each hemisphere by 9 to compute cells labeled per hour, and then multiplied by 24 to calculate new neurons per day.

### Neuron asymmetry index

**Figure 3:**
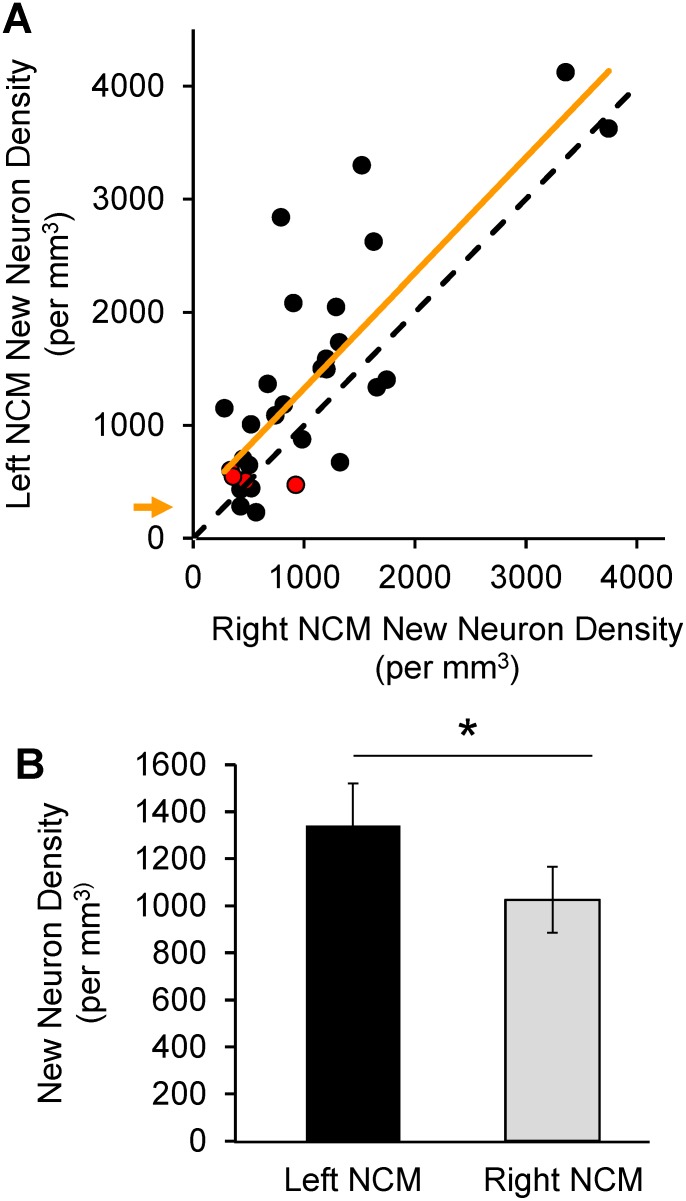
Hemispheric asymmetry in new neurons. (**A**) New neuron densities are significantly correlated between the hemispheres across birds. Points above the dashed identity line are birds with higher numbers of new neurons in left than right NCM. Best-fit regression (solid orange line) intercepts the Y-axis at 283.6 (orange arrow), indicating higher neuron density on the left (n = 31; r^2^ = 0.644; P<0.001). Red symbols are computer-tutored birds. (**B**) 1-month-old new neuron densities in NCM differ between the hemispheres. Mean new neuron density/mm^3^ was higher in the left NCM than the right NCM (Left = 1340.85±179.04 SEM, Right = 1025.31±139.92 SEM; paired t-test; n = 31, t = 2.999, P = 0.005, two-tailed).



The density of new neurons was strongly correlated between the hemispheres across birds (Fig. 3A), suggesting that neuronal incorporation in one hemisphere was associated with neuronal incorporation in the other, even though individual birds varied in absolute rates of neuronal incorporation. Therefore, to evaluate the hemispheric difference in new neuron densities within NCM, normalized for individual differences in overall new neuron numbers, we calculated a new Neuron Asymmetry Index (NAI) for each bird. To do this, we computed the difference between new neuron densities per mm^3^ in left and right NCM and divided this value by the density of new neurons in combined left and right NCM:

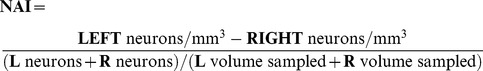



For this index, negative values indicate higher new neuron density on the right; positive values indicate a higher new neuron density on the left.

### New neuron nuclear diameters

New neuron counts are affected by the size of the neuron. Therefore, to determine whether there were lateral differences in new neuron nucleus size, we compared nuclear diameters of a sample of BrdU-labeled neurons (n = 10 cells per hemisphere per bird, n = 21 birds) between left and right NCM using Neurolucida to measure the longest diameter across the cell nucleus. Cells were selected by sampling the first 10 BrdU+ cells encountered with a standardized search criterion across hemispheres and birds (section 1–10, left to right, top to bottom, etc.) in successive sections of NCM.

### Neuron packing density

The packing density of all NeuN+ neurons (including BrdU+ and BrdU– neurons) was measured in a sample of 15 birds (chosen arbitrarily, blind to other data sets) in 2 NCM sections of each hemisphere matched for medial-lateral position across hemispheres and birds using field L and the caudomedial mesopallium (CMM) as landmarks. We counted all NeuN+ cells that had a visible nucleus in a 300 µm^2^ box placed in the center of NCM.

### Song recordings and analysis

Songs were recorded from 17 birds raised in family cages and 4 computer-tutored birds (n = 21). Two blind observers determined Similarity Indices (SI) between the subject bird’s song and that of his tutor using SAP 2011 software [43] and the average of the two scores was used in further analyses. Song stereotypy scores were computed by performing a similarity comparison among the song exemplars from each individual using SAP [37], [44]. Stereotypy scores were compared with SI, and also with new neuron numbers in each hemispheres, both hemispheres combined, as well as with the Neuron Asymmetry Index. Finally, SAP was used to compute measures of the following song features: Wiener entropy, goodness of pitch, pitch and frequency modulation. We also compared these features with measures of new neurons in each hemisphere, both hemispheres combined, and with the Neuron Asymmetry Index.

### Neurophysiological recordings in NCM

In preparation for neurophysiological experiments, birds were anesthetized either with isoflurane in oxygen (2%, Aerrane, Baxter, Deerfield, IL, USA) or Nembutal (50–55 mg/kg, Abbot Laboratories, North Chicago, IL), and surgically implanted with a head-fixation pin and recording chamber over the auditory forebrain using dental cement (Dentsply Caulk, Milford, DE). The electrophysiological recording was carried out at least 24 h after surgery to ensure complete recovery and elimination of the anesthetic.

Testing occurred inside a large acoustically isolated sound booth (IAC, Bronx, NY). The awake bird was restrained in a custom made body tube, and the head-fixation pin was clamped to a stereotaxic frame. A motorized micro-drive (Eckhorn, Thomas Recording, Giessen, Germany) was used to independently advance 16 micro-electrodes (quartz platinum/tungsten; impedance 1–3 megohms) into NCM. Eight electrodes were placed symmetrically within both left and right hemispheres. Extracellular multi-unit activity was simultaneously recorded from all 16 electrodes, amplified (total gain: 19,000), bandpass filtered (0.5–5 kHz) and digitized (25 kHz per channel) for further analysis as previously described [12]. Specialized software (Spike 2, Version 7, CED, Cambridge, UK) was used to deliver sound stimuli and record neural activity.

To measure the effect of previous song exposure on electrophysiological responses to songs (a measure of neuronal “memory”), the birds heard playbacks of 8 novel conspecific songs (200 repetitions, blocked, ISI 8 s) after recovery from anesthesia, 20 h prior to electrophysiological recording. Auditory responses were then recorded to these 8 now Familiar (F) songs and to 8 new Novel (N) songs (25 repetitions each in shuffled order). For assessment of auditory tuning properties at the recording sites, the birds also heard sets of artificial sounds: pure tones and band-limited noise (0.5–5 kHz in 0.25 kHz steps; 5 repetitions, ISI 6 s). All stimuli were presented free-field from a speaker located at a distance of 0.45 m directly in front of the subject at an average amplitude of 75 dB SPL (“A” scale).

### Analysis of neuronal responses to auditory stimuli

The multi-unit activity recorded with our microelectrodes and filter settings consisted of the action potentials of a small number (5–15) of single units near the microelectrode tip. Neural responses to song stimuli were quantified for each electrode as the difference between the root-mean-square voltage values (RMS) obtained during a response window (from stimulus onset to offset plus 100 ms) and the RMS during a control window on each trial (500 ms immediately prior to stimulus onset). Subtraction of the control RMS removes the contribution of ongoing baseline activity and recording noise. To compute the RMS, each digitized value was squared, the mean of these squares over the response interval was computed, and the square root of that mean was taken. This provided a method of rectifying the multi-unit activity and computing its average power. The RMS responses on trials 2–6 were averaged to compute absolute response magnitudes (ARMs) for each stimulus, as previously described [12].

To quantify the strength of the neuronal “memory” for stimuli heard 20 h earlier, the ARMs for all the stimuli of each type (Novel or Familiar) at each recording site were averaged and used to compute the Relative Response Strength (RRS) as the difference between ARMs for Novel and Familiar songs, normalized to their mean.



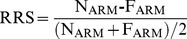



Auditory responses in NCM show stimulus-specific adaptation: responses to repetition of a given song stimulus decrease with repeated presentation of that stimulus, even if other stimuli intervene [45]. As a result, the ARMs for Novel songs are higher than those for Familiar songs, and thus the RRS provides a measure of the strength of familiarity. Positive values indicate overall higher responses to novel stimuli; negative values indicate overall higher responses to the familiar stimuli; values near zero indicate similar responses to both novel and familiar stimuli. For each bird, the RRS values were averaged across all sites in each hemisphere to produce a mean RRS value for each hemisphere.

### General statistics

To estimate the relationship between the numbers of new neurons counted and our experimental variables (Similarity Index, Relative Response Strength) we used a regression analysis. For comparisons between the hemispheres, we used the paired t-test, two-tailed. For all tests, the criterion for significance was set at P<0.05.

## Results

### Hemispheric asymmetry in new neurons

We measured new neurons bilaterally in the songbird caudomedial nidopallium (NCM; Fig. 2A), a telencephalic auditory area that responds differentially to familiar and unfamiliar conspecific songs, suggesting a role in storing “neuronal memories” of conspecific songs [31], [45], [46], [47]. We first asked whether the numbers of new neurons in NCM differ between the hemispheres by injecting adult male zebra finches with bromodeoxyuridine (BrdU) 3x/day over a 3 day period to label new cells and sacrificing the birds 31±2 days after the first BrdU injection to count new neurons co-labeled with antibodies to BrdU and the neuronal markers Hu or NeuN (Fig. 2B, as in [39]). There were no statistical differences in the Neuron Asymmetry Index between Hu+BrdU+ and NeuN+BrdU+ labeled cell counts; therefore, we combined the values for these cell groups for all analyses, except where specified.

The majority (22/31 = 71%) of the birds had more new neurons on the left than the right. Although the density of new neurons was strongly correlated between the hemispheres (n = 31; r^2^ = 0.644; P<0.001), the regression line between the two hemispheres showed a Y intercept at 283.6, corresponding to the higher new neuron density on the left across the population (Fig. 3A). A regression intercept at zero would indicate equal numbers of new neurons in each hemisphere. Across all birds, we found a significant hemispheric asymmetry, with higher mean new neuron density/mm^3^ in the left than in the right NCM (left = 1340.85±179.04 SEM, right = 1025.31±139.92 SEM; paired t-test; n = 31, t = 2.999, P = 0.005, two-tailed; Fig. 3B). This lateral difference represents ∼27% more new neurons labeled on the left side (see *Materials and Methods* for calculations). If we assume that new neurons are incorporated at a steady rate, the mean number of new neurons added to the left and right hemispheres was approximately 1709 and 1307 cells/mm^3^ per day, respectively (see *Materials and Methods* for calculations).

Nuclear diameters decrease with neuron age in HVC between ages 30 d and 240 d after cell birth dating [48]. Likewise, in caudal nidopallium (NC), BrdU-labeled 1-month-old neuron nuclear diameters were found to be “at the upper end of the distribution” of all non-birth dated NC neurons [49], suggesting that new neuron diameters decrease with cell age in NC as well. Therefore, we compared nuclear diameters of a sample of BrdU-labeled neurons (n = 10 cells per hemisphere per bird) between left and right NCM to determine whether our differences in cell counts reflected differences in neuron maturation between sides. If so, smaller nuclear diameters may artificially decrease our cell counts. We found no difference in mean nuclear diameters of BrdU+ neurons between left and right NCM (paired t-test, n = 21, t = 0.908, P = 0.380, two-tailed).

To evaluate whether differences in new neuron addition between hemispheres are cumulative, we measured neuronal packing density (not restricted to BrdU+ neurons) in left and right hemispheres in a subset of birds. We did not find any differences in the density of NeuN+ cells between hemispheres (paired t-test, n = 15, t = 0.452, P = 0.658, two-tailed).

### Asymmetry in new neuron incorporation is related to song copying

**Figure 4:**
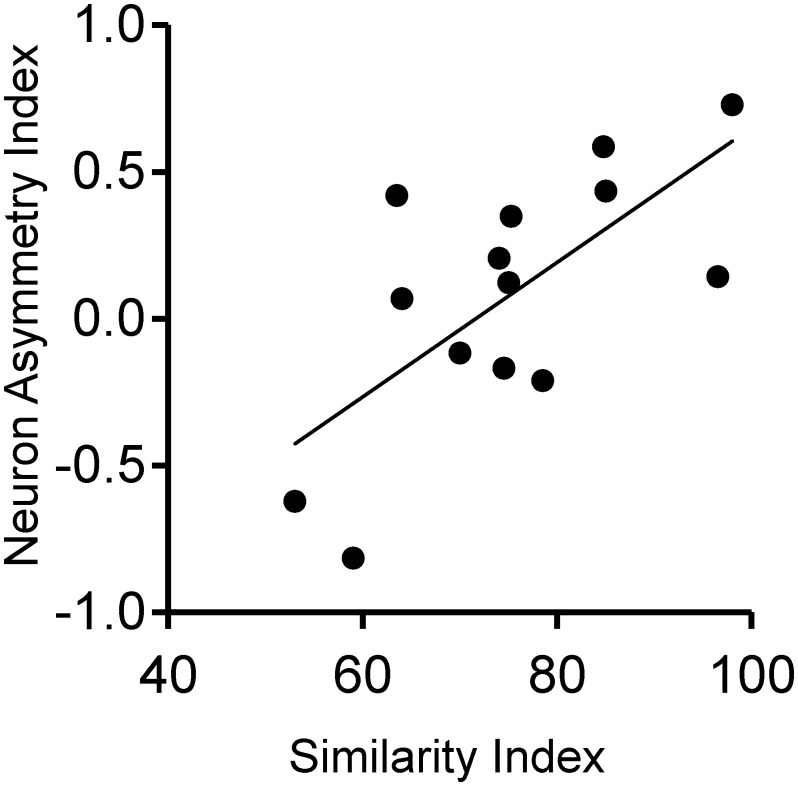
Hemispheric asymmetry in new neuron density is correlated with individual success in tutor song copying. Higher new neuron density on the left than the right measured by a positive Neuron Asymmetry Index (NAI) is correlated with higher quality song imitation measured by the Similarity Index (n = 14; r^2^ = 0.467; P = 0.007).

Next we tested the hypothesis that adult neuron incorporation is related to differences in learning among birds. Individual success in tutor song copying was quantified using an established Similarity Index (SI, Fig. 4) that measures how similar each bird’s own song is to its tutor’s song [31], [43]. When considered individually, neither hemisphere showed a significant correlation between the SI and new neuron density in NCM (n = 14; left: r^2^ = 0.073, P = 0.349; right: r^2^ = 0.004, P = 0.840). In addition, there was no correlation between SI and new neurons per mm^3^ across both hemispheres (r^2^ = 0.003, P = 0.817). However, when this measure of imitation quality was compared to the difference in new neuron density between hemispheres, calculated as a normalized new Neuron Asymmetry Index (NAI, see *Materials and Methods*), NAI and SI were significantly correlated (Fig. 4; n = 14, r^2^ = 0.467, P = 0.007). This suggests that the relative level of new neuron incorporation between hemispheres can predict individual differences in vocal learning.

Because the values for SI and NAI were averaged among brothers for the data reported, we also assessed whether the same results were found when values from each bird, not considering relatedness, were compared (total n = 21 birds with song recordings and new neuron counts). We found a significant correlation between the SI and the NAI when all birds were used independently (n = 21; r^2^ = 0.233, P = 0.027). Again, when all birds were considered individually, neither hemisphere showed a significant correlation between the quality of song imitation as measured by SI and new neuron density in NCM, although there was a trend toward significance in the left hemisphere only (n = 21; left: r^2^ = 0.155, P = 0.077; right: r^2^ = 0.055, P = 0.305). There was no correlation between SI and total new neurons across both hemispheres (n = 21; r^2^ = 0.116, P = 0.131). In addition, we performed a third analysis limited to the 11 sibling groups who had live tutors; again, we found a significant correlation between SI and NAI (n_live tutors_ = 11; r^2^ = 0.583, P = 0.006) and no correlations between SI and total new neurons in either or both hemispheres (P>0.05 for all).

We also quantified individual song stereotypy in relation to NAI because other work has demonstrated a positive correlation between numbers of new neurons in the song motor nucleus HVC and syllable variability [44]. The stereotypy score of songs produced for each bird as a measure of song maturity (see *Materials and Methods*; [37]) was not correlated with the NAI or with new neuron counts in either or both hemisphere(s) (P>0.05 for all). None of the other song features measured (Wiener entropy, goodness of pitch, pitch and frequency modulation) showed a correlation with new neurons in either hemisphere, both hemispheres, or NAI.

**Figure 5:**
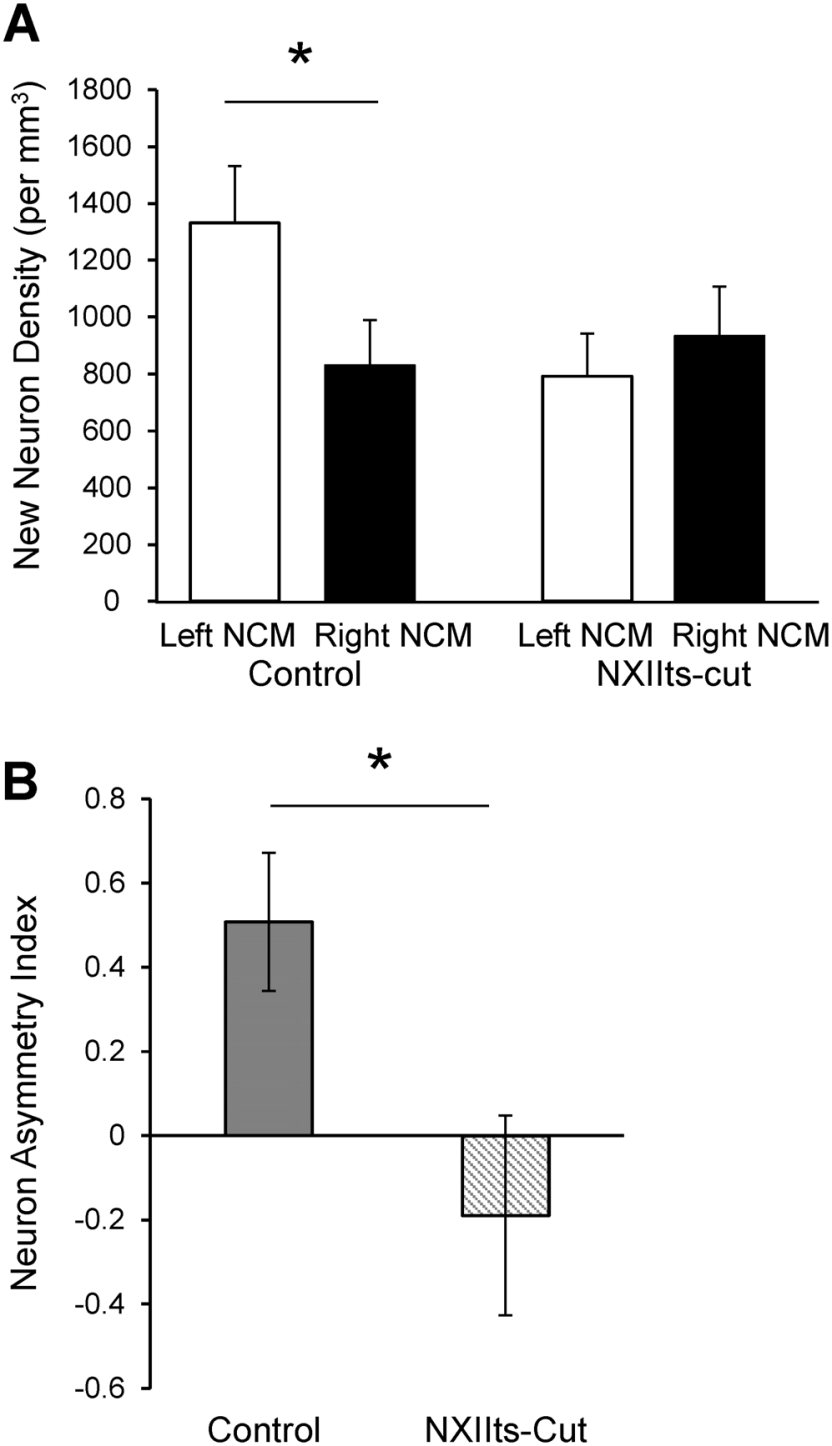
Vocal nerve section alters new neuron lateralization. (A) New neuron densities in NCM differ between the hemispheres in a subset of control birds (Left = 1331±200 SEM, Right = 831±157 SEM; paired t-test; n = 7, t = 3.500, P = 0.013, two-tailed) but not in NXIIts-cut birds (Left = 793±149 SEM, Right = 933±174 SEM; paired t-test; n = 13, t = −0.545, P = 0.595 two-tailed). (B) The Neuron Asymmetry Index (NAI) shows a left-side bias in control birds (0.513±0.163 SEM, n = 7), significantly different from NAI in NXIIts-cut birds processed simultaneously (–0.277±0.253 SEM, n = 13; unpaired t-test, t = 2.133, P = 0.045).

The correlation between NAI and SI quality we observed does not reveal the causal relationship between these variables. To address this, we tested whether feedback from the bird’s own song may contribute to maintaining lateralized new neuron incorporation. We assessed new neuron lateralization in a set of birds whose song production was degraded by unilaterally sectioning the right or left tracheosyringeal nerve (NXIIts). This treatment affected vocal production, leading to a change in auditory feedback during vocalization, and it may also have affected somatosensory feedback [25], [50], [51], [52]. When we processed brains from these birds together with brains from intact control birds, NXIIts-cut birds did not show any hemispheric asymmetry in new neuron incorporation per mm^3^ (left = 793±149 SEM, right = 933±174 SEM; paired t-test; n = 13, t = −0.545, P = 0.595, two-tailed; Fig. 5A), while the controls showed the expected higher number of new neurons in left than right hemisphere (left = 1331±200 SEM, right = 831±157 SEM; paired t-test; n = 7, t = 3.500, P = 0.013, two-tailed). Moreover, the NAI for controls was significantly different from that of the NXIIts-cut group (control: 0.513±0.163 SEM, n = 7; NXIIts-cut birds: −0.277±0.253 SEM, n = 13; unpaired t-test, t = 2.133, P = 0.045; Fig. 5B).

### Asymmetry in new neuron incorporation is related to neuronal memory for song

We then tested whether asymmetrical neuron incorporation also corresponds to the formation of new neuronal memories in NCM of adult birds using an established neurophysiological approach [12], [31]. As described above, neurons in NCM respond differentially to familiar and unfamiliar songs; thus this property can be thought of as a neuronal memory for songs the bird has previously heard [45], [46]. For example, a record of the tutor song (acquired in the juvenile period 30 days earlier) can be detected in the patterns of song-evoked activity and adaptation in adult NCM and the strength of this neuronal memory is correlated with the quality of song imitation [31], [53].

**Figure 6:**
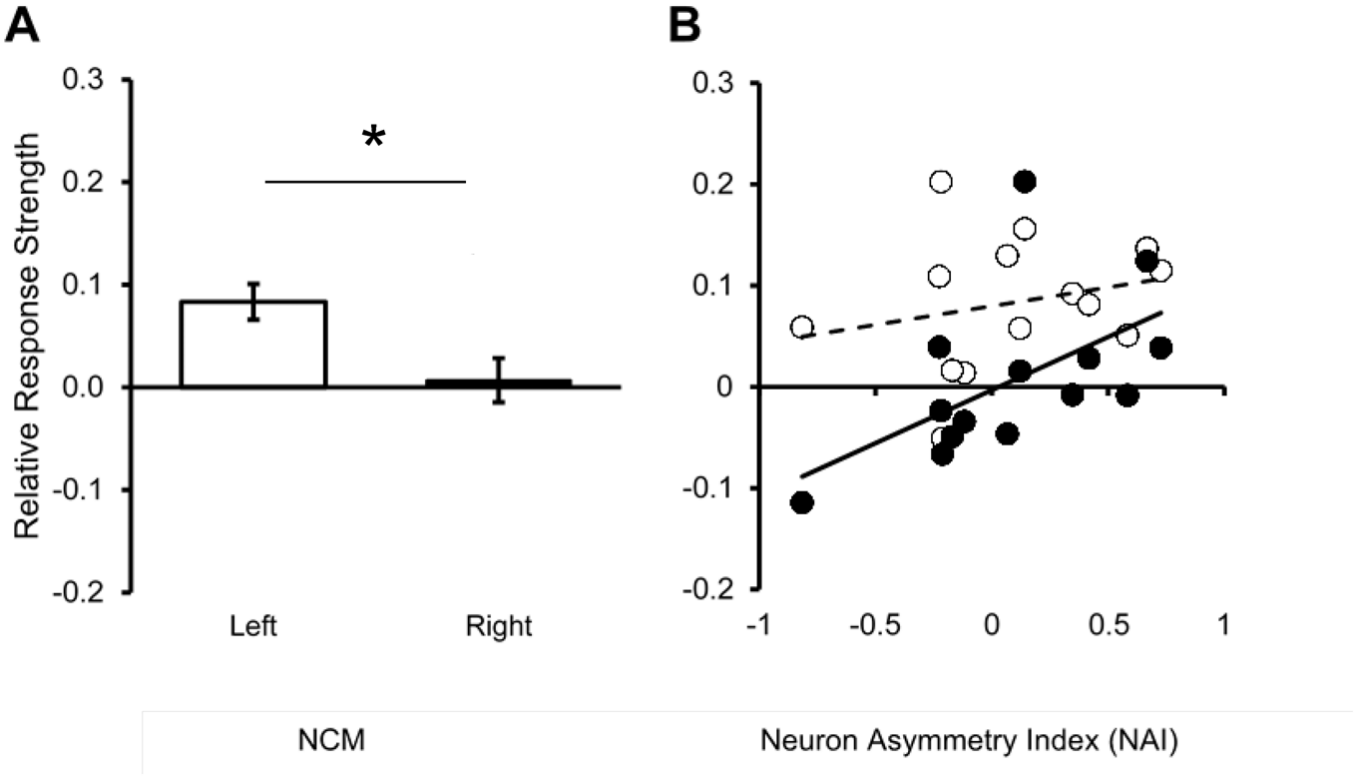
Hemispheric difference in neuronal memory for songs heard in adulthood. (**A**) Neuronal memory for songs heard 20 h earlier, measured as the Relative Response Strength (RRS) is significantly higher on the left than the right (n = 14; Left = 0.083±0.018 SEM; Right = 0.007±0.021 SEM; paired t-test; t = 3.95, P = 0.002, two-tailed). (**B**) Birds with the highest Neuron Asymmetry Index (NAI) indicating relatively more new neurons on the left side, had the highest RRS on the right. When responses in each hemisphere were compared to new neuron lateralization, NAI and RRS were not correlated in left NCM (n = 14; r^2^ = 0.058; P = 0.406; white symbols). In contrast, RRS in right NCM had a wider range and was positively correlated with the NAI (n = 14; r^2^ = 0.312, P = 0.038; black symbols).

To compare individual differences in the strength of neuronal memories for songs, isolated adult birds were exposed to multiple repetitions of the unique songs of 8 unfamiliar zebra finches. Twenty hours later, extracellular multiunit recordings were made at 16 sites bilaterally in NCM during playback of the songs previously heard (now Familiar), together with a set of Novel songs used to normalize individual differences in responsivity. The strength of 20-hr memories for Familiar songs was calculated as the normalized difference in response magnitude to Familiar versus Novel song stimuli, providing a Relative Response Strength (RRS, see *Materials and Methods*), a measure of neuronal memory. Across birds, RRS was higher in the left than the right hemisphere (n = 14; left = 0.083±0.018 SEM; right = 0.007±0.021 SEM; paired t-test; t = 3.95, P = 0.002, two-tailed; Fig. 6A). However, there was no correlation between left-side RRS and new neuron number on the left or between right-side RRS and new neuron number on the right (n = 14; left r^2^ = 0.045, P = 0.465; right r^2^ = 0.002, P = 0.895), suggesting that the RRS index did not reflect the numbers of 1-month-old neurons on either side.

When responses in each hemisphere were compared to new neuron lateralization, RRS values in left NCM were not correlated with NAI (n = 14; r^2^ = 0.058; P = 0.406; Fig. 6B). In contrast, RRS in right NCM showed greater individual differences and was positively correlated with NAI (n = 14; r^2^ = 0.312, P = 0.038). Thus, RRS was higher in the left hemisphere than the right, while birds with higher NAI (relatively more new neurons on the left side) had relatively high RRS (neuronal memory strength) on the right side. Thus right hemisphere participation in memory for songs heard 20 h earlier appears to be stronger when relatively more new neurons are incorporated into left NCM and fewer into right NCM.

## Discussion

Together, these results show that more new neurons were added to left NCM than right NCM of adult birds when new cells were assessed 30 days after cell birth dating. Individual differences in the degree of asymmetry in new neuron incorporation were correlated with both the quality of song imitation and the strength of neuronal memories for recently heard conspecific songs. The density of new neurons in the left NCM, but not right NCM, showed a trend toward a significant correlation with song learning, but not with our measures of memory. These findings suggest that the relative difference in new neurons between the hemispheres is an important factor in song learning and memory processes. However, these relationships do not show the direction of causation [54], i.e., whether birds with a greater initial asymmetry in new neurons produce better imitations or whether singing a good song imitation drives greater hemispheric asymmetry. The latter idea was supported when we found no asymmetry in new neurons in birds with experimentally-impaired song quality.

One possible explanation for the pattern of results is that birds that sing better imitations experience a better match between hearing their own song and their internal memory of the tutor song, and successful matching increases neuronal survival in the left hemisphere relative to the right. Immediate early gene (zenk) expression in response to playback of the tutor’s song is higher in left than in right NCM during juvenile song learning, suggesting a stronger memory specifically for the tutor song in the left hemisphere [13]. Thus, the evaluation of the developing (and mature) song in comparison with the tutor song memory may be left-side lateralized, and perhaps mediated via neurotrophins such as BDNF [55], local synthesis of estradiol in NCM in response to hearing song [56], [57], or by neuromodulators involved in reward and reinforcement, such as dopamine and opioid peptides [58], [59], [60], [61]. These transmitter types have been associated with regulating neurogenesis and new neuron survival [60], [62], [63], [64] and have been shown to function in a hemisphere-specific manner [65], [66], [67] although their role in NCM has not been determined [68]. Regardless of the specific trophic mechanism regulating new neuron survival, it must be turned on in an asymmetric way, either because producing a good imitation engages the hemispheres differentially (perhaps through a matching mechanism), or because the trophic process induced by a good imitation is itself asymmetrical.

Our data showing an absence of new neuron lateralization after nerve cuts that alter song support a possible role for song-matching in asymmetric new neuron incorporation. However, we cannot rule out other interpretations. For example, denervation can lead to motor neuron death and might produce retrograde degeneration with transynaptic effects that reach NCM. Earlier work showed that unilateral syringeal denervation in juveniles during song learning resulted in a transitory increase in new neurons in the contralateral song system nucleus HVC, which is part of the song production pathway [14], [15]. Because the vocal motor pathway is ipsilateral, this effect was thought to involve compensatory effects, and similar processes cannot be ruled out in our sample.

Likewise, our data cannot rule out the possibility that some birds have more asymmetric neurogenesis favoring the left side independent of song learning experience and this asymmetry enables them to be better learners; thus they form more robust memories of both the tutor song and of songs newly heard in adulthood. However, in mammalian studies that found a relationship between the number of new neurons in the hippocampus (examined within one hemisphere) and learning, the number of surviving neurons was shown to reflect the learning process itself and was not attributable to inherent differences in “smarter” animals [69], [70].

Nonetheless, these possible explanations are not exclusive, since initial asymmetries in neuron incorporation during development might result in better song imitation, which in turn might maintain or enhance the asymmetry through matching-driven trophic factor release. Further experiments that manipulate neuronal incorporation independently in the two hemispheres while also manipulating the quality of imitation are needed to clarify the contribution of these possible mechanisms in juveniles and adults.

Our observations are limited to one time point in the life history of new neurons in NCM. New neurons in the caudal nidopallium (of which NCM is the medial region) have variable lifespans, which differ along a rostro-caudal dimension [49], [71]. Barnea et al. [49] proposed that if new neurons encode experience, they may also provide a time stamp for such experience, which may be represented by how long a given population of neurons survive. In this way, regions of the caudal nidopallium with rapid neuronal turnover would correspond to storage of recent events whereas regions that undergo slower turnover would be expected to retain older memories. Neuronal packing density in NCM did not differ between hemispheres in our sample, suggesting that our counts of new neurons reflect the rate of neuronal replacement [49], [72], [73], as opposed to cumulative addition [38]. If so, we may be observing a population of shorter-lived neurons in left NCM than right NCM – and this population may contribute to plasticity during song learning. Alternatively, our sample size may be insufficient to see increased packing density due to cumulative addition of new neurons.

It is significant that the *relative* numbers of new neurons between the hemispheres predict imitation quality and auditory memory whereas the absolute numbers in either hemisphere (or both) do not. A combination of high numbers of new neurons in left NCM with low numbers in right NCM results in the best song learning. Furthermore, birds with negative values of neural lateralization (the minority with more new neurons on the right) had the lowest measures of song learning and memory suggesting that left hemisphere lateralization specifically, and not a lateral difference *per se*, predicts better learning and memory. This finding could be evidence of a competitive process between the sides and suggests a behavioral advantage to hemispheric specialization, which may simply improve processing capacity through a division of labor. For example, behavioral work in birds with unilateral lesions of the auditory thalamus showed different deficits in auditory song discrimination depending on the side of the lesion, suggesting that the two hemispheres process conspecific songs differently [24]. However, the correlations between learning and memory and the *difference* in neuron incorporation suggests that lateralization may act to segregate processing streams to avoid conflict and/or that comparison of activity between the hemispheres may contribute to enhanced discrimination and memory processes. Likewise, the degree of cerebral lateralization has been shown to predict cognitive performance in a visual discrimination task in pigeons [74] and auditory discrimination for speech sounds in human subjects [75].

Birds do not have a corpus callosum, so any contralateral effects on learning and memory reflected in neuron incorporation are likely to be mediated at the brainstem or midbrain level [76]. However, interhemispheric integration of hemisphere-specific information has been shown to improve visual task performance in pigeons, demonstrating that hemispheric specialization and functional interhemispheric interactions do not require a corpus callosum [77], [78]. In mammals, there is evidence of lateral differences in brainstem auditory processing that do not depend on callosal connections [6]. Thus, the basic mechanisms of asymmetry, which probably provide the initial basis for lateralization of complex functions, may well be shared in the vertebrate lineage [4], [7], [79].

A combination of innate factors and/or very early experience might set up lateral processing differences (e.g. [77]), which then become reinforced through further experience and hearing self-vocalizations. For example, our earlier work showed differences in responses to song playback between left and right NCM that support the idea that the left side forms more lasting memories for particular classes of sounds during early auditory experience [12]. At the same time, the right hemisphere may subserve other auditory functions not yet characterized, possibly analogous to the complementary contributions to speech processing of the two sides of the human brain [18], [20], [80]. In zebra finches, vocal production is thought to be right-side dominant, based on lesions of the song control pathway [81]. Future work will be required to elucidate the relationship between this asymmetric contribution to vocal production and the opposite asymmetry in neuronal incorporation we document here. Indeed, the relationship between lateralized processing and lateralized production for human speech is not well understood [82].

In the mammalian brain, new neurons are incorporated primarily into the hippocampus, olfactory bulb, and hypothalamus [83], [84]. Whereas there are numerous reports of structural and functional hemispheric asymmetries in these regions [8], [79], [85], thorough investigation of lateral differences in neuron incorporation in the mammalian brain has not been reported. Hemispheric asymmetry is a factor in numerous neurological diseases, including Parkinson’s Disease [86], Alzheimer’s Disease [87], and schizophrenia [88]. Furthermore, decreased asymmetry in frontal lobe activity is associated with cognitive decline in the aging brain in humans [89], supporting the idea of a role of hemispheric specialization in cognitive task performance [90]. This underscores the importance of understanding lateralized neurogenesis not only for advancing knowledge about healthy brain function, but also to provide new ideas for how controlled neurogenesis might be used for brain repair [91], [92]. On a basic level, the asymmetric pattern of neuronal incorporation we have documented in the auditory forebrain of a songbird, where ongoing adult neuron incorporation may provide a window on developmental processes, contributes another way of thinking about how the addition of new neurons to existing neural pathways could contribute to behavior, and may shed light on the origins of lateralized functions in the brain.
